# Deoxynivalenol affects cell metabolism in vivo and inhibits protein synthesis in IPEC-1 cells

**DOI:** 10.1007/s12550-023-00489-z

**Published:** 2023-05-31

**Authors:** Constanze Nossol, Peter Landgraf, Anikó Barta-Böszörmenyi, Stefan Kahlert, Jeannette Kluess, Berend Isermann, Oliver Stork, Daniela C. Dieterich, Sven Dänicke, H.-J. Rothkötter

**Affiliations:** 1 Institute of Anatomy, Medical Faculty, Otto-von-Guericke-University Magdeburg, Leipziger Strasse 44, Magdeburg, 39120 Germany; 2 Institute of Pharmacology and Toxicology, Medical Faculty, Otto-von-Guericke-University Magdeburg, Leipziger Strasse 44, Magdeburg, 39120 Germany; 3 Friedrich-Loeffler Institute, Braunschweig, 38116 Germany; 4 Institute of Laboratory Medicine, Clinical Chemistry and Molecular Diagnostics, University of Leipzig, Leipzig, 04103 Germany; 5 Deparment of Genetics and Molecular Neurobiology, Institute of Biology, Otto-von-Guericke-University Magdeburg, Leipziger Strasse 44, Magdeburg, 39120 Germany

**Keywords:** IPEC-1, Metabolism, ATP, Glucose, Lactate, Dexynivalenol

## Abstract

Deoxynivalenol is present in forage crops in concentrations that endanger animal welfare but is also found in cereal-based food. The amphipathic nature of mycotoxins allows them to cross the cell membrane and interacts with different cell organelles such as mitochondria and ribosomes. In our study, we investigated the gene expression of several genes in vivo and in vitro that are related to the metabolism. We observed a significantly higher COX5B and MHCII expression in enterocytes of DON-fed pigs compared to CON-fed pigs and a marked increase in GAPDH and SLC7A11 in DON-fed pigs, but we could not confirm this in vitro in IPEC-1. In vitro, functional metabolic analyses were performed with a seahorse analyzer. A significant increase of non-mitochondrial respiration was observed in all DON-treatment groups (50–2000 ng/mL). The oxygen consumption of cells, which were cultured on membranes, was examined with a fiber-glass electrode. Here, we found significantly lower values for DON 200- and DON 2000-treatment group. The effect on ribosomes was investigated using biorthogonal non-canonical amino acid tagging (BONCAT) to tag newly synthesized proteins. A significantly reduced amount was found in almost all DON-treatment groups. Our findings clearly show that apical and basolateral DON-treatment of epithelial cell layer results in decreasing amounts of newly synthesized proteins. Furthermore, our study shows that DON affects enterocyte metabolism in vivo and in vitro*.*

## Introduction

The fungus genus Fusarium produces a broad spectrum of metabolites, including mycotoxins like deoxynivalenol (DON). Despite minimization strategies, DON is present in forage crops in concentrations that endanger animal welfare and pigs are particularly sensitive to them (Pestka 2007). A study showed that DON causes an economical loss in the animal production due to vomiting, reduced feed intake and growth (Rotter et al. 1996). DON and NIV caused growth retardation in mice (Ryu et al. 1988; Ohtsuboet al. 1989), increased postnatal mortality (Khera et al. 1984) and increased susceptibility of mice to infections (Tryphonas et al. 1986). The oral exposure to DON induces different proinflammatory cytokines and chemokines in mice that can be expressed in liver, spleen, kidney, and lung (Kinser et al. 2004, 2005; Pestka and Amuzie 2008). In vitro, Pestka and He demonstrated an upregulation of pro-inflammatory cytokines such as IL-6, TNFα and IL-1β on RNA-level (Pestka 2010; He et al. 2013). Additionally, Nossol and colleagues found a higher number of pores in the basement membrane in DON-fed pigs and consequently a higher number of CD16^+^ positive cells in the jejunal epithelium (Nossol et al. 2013). DON has complex effects on organism and belongs to type II of trichothecenes, which inhibit the elongation-termination step (Ueno 1985) and, therefore, it has an impact on protein biosynthesis. In an in vivo study, Dänicke and co-workers showed a significantly reduced protein synthesis (fractional synthesis rate (FSR)) in kidneys, spleen, and ileum of DON-exposed pigs but jejunum and jejunal mucosa cells were not affected (Dänicke et al. 2006). This could be confirmed with an in vitro study of Nossol and co-workers (Nossol et al. 2018). They demonstrated, that IPEC-J2 cells (intestinal porcine epithelial cells; jejunal origin) showed neither an increase nor a decrease in protein biosynthesis at low DON concentrations (50 ng/mL apical or basolateral; 200 ng/mL apical) but DON-treated cells (200 ng/mL basolateral, 2000 ng/mL apical and basolateral) resulted in an increased protein biosynthesis (Nossol et al. 2018). Furthermore, Diesing and colleagues examined the effect of DON on RNA expression in IPEC-J2 cells. Significantly regulated KEGG-pathways were found to be carbohydrate metabolism, energy metabolism, transcription and translation, folding, sorting and degradation of proteins (Diesing et al. 2012). So, we addressed the question, if there is a decrease in protein synthesis rate of IPEC-1 cells (a mixture of ileal and jejunal cells) as observed in vivo? Therefore, we used biorthogonal non-canonical amino acid tagging (BONCAT) in which azidohomoalanine is used as a surrogate for methionine in order to tag newly synthesized proteins in our cell line IPEC-1 (Dieterich et al. 2007).

The amphipathic nature of trichothecenes allows them to cross the cell membrane and interact with different cell organelles such as endoplasmatic reticulum (Yang et al. 2000) and mitochondria (Pace 1983). Pace and co-worker investigated in vivo and in vitro the effect of T2-toxin on electron transport in rat mitochondria. They showed an inhibited oxygen consumption through T2-toxin (2.2 mM) (Pace 1983).

In addition, we hypothesize that there are also changes in the metabolism of the enterocytes. To answer this question, we studied oxygen consumption, glucose uptake, lactate production and ATP production of IPEC-1 cells. DON interacts with epithelial cells on the apical site during intestinal passage and absorption, but detectable DON concentrations were also found in the blood serum (Dänicke et al. 2004). For this reason, we have also studied low concentrations of DON (50 ng/mL and 200 ng/mL). When in vitro effects of the mycotoxins were compared with the results from the whole animal they did not match. For example, it may be the case that a mycotoxin is a weak inhibitor of the protein biosynthesis in vitro but behaves like T2-toxin in animals. To investigate this discrepancy, we used laser dissection to harvest the epithelium and isolate RNA of the enterocytes. This enables us to compare in vivo RNA data with our in vitro RNA data.

## Material and methods

### Cell culture

Intestinal porcine epithelial cells (IPEC-1 ACC 701; Steube et al. 2012) were regularly tested and found to be free of mycoplasma contamination (Venor GeM Mycoplasma Detection Kit; Minerva Biolabs, Berlin, Germany). In all experiments, cells were seeded with a density of 1*10^5^/cm^2^ on permeable support (ThinCerts; pore size: 1 µm; polyester, Greiner bio-one, Germany). DMEM/HAMs F12 supplemented with 5% fetal bovine serum (FBS), ITS (insulin-transferrin-selenium) 5 mL/500 mL cell culture medium, 16 mM 4-(2-hydroxyethyl)-1-piperazineethansulfonic acid (HEPES) (all: PAN-Biotech, Aidenbach, Germany), and 5 ng/mL epidermal growth factor (EGF, Biochrome, Berlin, Germany) were used as cell culture medium. Cells grew at 39 °C in an atmosphere of 5% CO_2_ and 95% relative humidity. The transepithelial electrical resistance was measured on day 10 of cell culture using a Millicell-TERS-electrode (Millipore, Berlin, Germany) and after DON-application.

### DON-application

Deoxynivalenol (DON; D0156; Sigma-Aldrich, Germany) was as diluted in absolute ethanol (99.6%; Roth, Germany) to 0.2 mg/mL stock solution and the respective working dilutions were prepared in cell culture medium. Two low DON concentrations of 50 ng/mL and 200 ng/mL and a high DON concentration of 2000 ng/mL were applied reflecting non-toxic and a toxic dosage as found in conventional cytotoxicity studies (Diesing et al. 2011; Nossol et al. 2013). A final concentration of 1% ethanol corresponding to the ethanol concentration of 2000 ng/mL DON solution was tested and results were not significantly different from control (Diesing et al. 2011).

### Pig experiment

Experiment and procedures were conducted according to the European Community regulations concerning the protection of experimental animals and the guidelines of the German Animal Welfare Act and were approved by the ethical committee of the Lower Saxony State Office for Consumer Protection and Food Safety (file number 33.4-42502-04-13/1274).

In our study, we investigated the effect of DON-contaminated feed on pigs. The whole study was accomplished using a total of 44 barrows (German Landrace, Mariensee, Germany). The animals had an initial mean body weight (BW) of 25.8 ± 3.7 kg and were divided into two feeding groups. The first group was chronically exposed to a diet which contains naturally DON-contaminated (DON-fed; 4 weeks) maize and the second group were fed with the same diet but without contaminated maize (CON-fed; 4 weeks). Each pig received 4.59 mg DON/kg feed per day. Further information about the pig experiment and surgery is found in Tesch et al. (Tesch et al. 2015). Gut samples were taken and immediately frozen in liquid nitrogen (− 80 °C).

### Laser-capture microdissection (LCM) and qPCR

RNAse-free membrane slides (Zeiss; Oberkochen, Germany), coated with 0.05% poly-L-lysine (Sigma St. Louis, USA) were used. All experiments were conducted with RNAse free material and using DMDC-aqua dest.

Porcine jejunum (CON-Fed and DON-fed; 5 animals per group) was embedded in tissue freezing medium (Leica, Wetzlar, Germany). Thin gut sections (10 µm) were prepared in a cryostat at − 18 °C, placed onto membrane glass slides and dried on a warming plate (2 min; 40 °C). In the next step, thin sections were fixed with − 20 °C pre-cooled 70% ethanol in DMDC-Aqua for 1 min, stained with Cresyl Violet (1. 1% Cresyl Violet solution in 50% ethanol/DMDC-Aqua, 4 °C; 1 min; 2. 70% ethanol-DMDC-Aqua, 2 min, 4 °C; 3. 100% ethanol, 2 min, 4 °C) and dried on a hot plate 40 °C. Areas of interest (enterocytes of the villus without basal membrane) were marked with freehand tool (× 10 magnification; 3 000 000 µm^2^/sample; Palm Robo 4.4, Zeiss, Oberkochen, Germany). In the next step, using the tool AutoLPC (Laser energy cutting: 50%/Focus: 83%; LPC: 83%/Focus: 81%) areas of interest were removed and placed into adhesive caps (Zeiss, Oberkochen, Germany). Furthermore, 350 µL RLTplus (+ ß-Mercaptoethanol) was added to the tube, incubated “up-side-down” for 30 min on ice and centrifuged for 5 min (RT; 12000 rpm). Supernatants were added to a “gDNA eliminator column” (30 s; 8000 × g). Samples were processed according to manufacturer’s instructions (RNeasy Plus Micro Kit, Qiagen, Hilden, Germany). Finally, RNeasy MinElute spin columns were eluted with 2 × 14 µL RNase-free water.

Quantitative PCR amplification was performed for all genes under following conditions using a qTower (Analytik Jena, Germany): 10 min at 45 °C for reverse transcription, 2 min at 95 °C (polymerase activation) followed by 40 cycles of 30 s at 95 °C and 60 s at optimal primer annealing temperature (Table 1). Melting curve analysis (50–95 °C) was used for assessing amplification specificity. The reaction volume of 10 µL contained 5 µL SensiFAST SYBR/No-ROX (2 × , One-Step-Mix, Bioline, Germany), 0.4 µL of the respective primers (10 pmol/µL), 2.9 µL nuclease free water, and 1 µL RNA (2 ng/µL).

**Table 1:** Used primer pairs

***Gene***	***Left (5′-3′)***	***Right (3′-5′)***	***Temperature [°C]***
**18S**	GCAATTATTCCCCATGAACG	GGCCTCACTAAACCATCCAA	56.5
**β-actin**	GATGAGATTGGCATGGCTTT	CACCTTCACCGTTCCAGTTT	58.3
**TXNIP**	AGCAGCCAAGAGAACAGAGA	TCCACGGACACAATACCCA	57.2
**COX5B**	TGATGAGGAGCAGGCGAC	GTCGGAGTCCATGGTTCCTT	59.4
**PHB**	TGAAAACTCTGCCCCTGTGA	TCTGCAGGACTCACATCTCG	57.3
**SLC7A11**	TAAATTTGGGTGCAATGTGATGT	TTGAAGCAACTAGAAGCATGACA	54.9
**GAPDH**	ACCCAGAAGACTGTGGATGG	TTGAGCTCAGGGATGACCT	59.5
**TRIC**	ACGTGGCGAAATACCCTATG	CTTCAAGACGGCCTGAACTT	56.5
**MHCII**	CTCATGCAATTCCGGTTTTCC	TGCCACGCTGACATTTACTG	59.4

The analysis comprised five animals per group with each sample in triplicates. The ddCt method was used for the calculation of differences in the gene expression (ratio = 2^−ddCt^) (Pfaffl 2001). The differences between the DON-fed samples and CON-fed were normalized to the individual expression of the geometric mean of the two housekeeping genes: ß-actin and 18S (Table 1).

### RNA isolation of IPEC-1 and qPCR

After withdrawal of apical and basolateral medium, cells were covered with TRIzol reagent (Invitrogen, Germany) as described by the manufactur’s protocol and scraped off the membrane. After adding chloroform to the cell lysate, supernatant was extracted and RNA was precipitated using isopropanol alcohol. Using 75% ethanol the RNA was purified and stored in RNA-free water peqGOLD (peqlab, Germany) at − 80 °C until further processing.

RNA obtained from five independently repeated experiments was used as template for qPCR. Each 1 µg of template RNA was subjected to reverse transcription with First Aid Reverse Transcription Reagents (Fermentas, Germany) essentially as described by the manufacturer with the supplied random hexamer primers in a ThermalCycler TC1 (Biometra, Germany). The resulting cDNA samples were used for qPCR amplification of the seven chosen genes.

Quantitative PCR amplification was performed for all genes under following conditions on an iCycler (BioRad, Germany): 5 min at 95 °C followed by 40 cycles of 30 s at 95 °C and 60 s at optimal primer annealing temperature (Table 1). Melting curve analysis (50–95 °C) was used for assessing amplification specificity. The reaction volume of 20 µL contained 10 µL Maxima Mastermix (2 × , Fermentas, Germany) with SYBR^®^ Green and Fluorescein as internal standard, 3 µL of the respective primers (2 pmol/µL), 2 µL nuclease-free water and 2 µL cDNA (60 ng/µL).

The analysis comprised five independent experiments with each sample in triplicate. The ddCt method was used for the calculation of differences in the gene expression (ratio = 2^−ddCt^) (Pfaffl 2001). The differences between the DON-treated samples and the untreated control in the relative quantification (rq) values were normalized to the individual expression of the geometric mean of the two housekeeping genes: ß-actin and 18S.

### Oxygen measurement

The oxygen uptake was measured with a Microx TX3 (Presens, Regensburg, Germany). Microx TX3 is a microfiber optic oxygen meter with a microsensor based on a 140 µm optical fiber. The sensor was calibrated according to the manufacturer’s instructions (manual; 2-point calibration). The oxygen content in the medium (apical from the cells was measured for 10 min. The oxygen uptake is given due to the difference between both values (sample value – blank value).

### Analysis of mitochondrial function

Cells were seeded with a density of 1*10^5^/cm^2^ (100 µL/well) on a plate (Agilent, Germany) and cultured as described as above. On day 7 DON was added at different concentrations: 0 ng/mL (CON), 50 ng/mL, 100 ng/mL, 200 ng/mL, 500 ng/mL, and 2000 ng/mL and cells were cultured for further 3 days. On day 10, a mitostress test was performed using a Seahorse Analyzer (Agilent Technologies). On the day before, a sensor cartridge had to be hydrated with 200 µL/well ampuwa^®^ and at the same time, 20 mL of calibrant (Agilent, USA) was stored overnight in the same incubator (39 °C; non-CO_2_). On the day of experiment, ampuwa was aspirated and calibrant was added (200 µL/well; pre-warmed, overnight). Furthermore, sensor cartridge was incubated for further 45 min (39 °C; non-CO_2_) before starting the experiment. In the next step, assay medium was prepared with the supplementation of 17.5 mM glucose, 1 mM pyruvate and 2.5 mM glutamine to the XF base medium (Agilent Technologies, USA). The pH was adjusted to 7.40 at 39 °C with 0.1 M NaOH and medium was sterile filtered. Stock solutions were produced according to manufacturer`s protocol which were used to prepare operating concentrations (oligomycin: 15 µM; FCCP: 20 µM; rotenone/antimycin A: 5 µM).

Cell plate was washed with assay medium and 500 µL of the assay medium was added to the wells and plate was incubated for 45 min at 39 °C/non-CO_2_. During incubation, ports of the sensor cartridge were filled (port A: 56 µL, port B: 62 µL; port: C: 69 µL) with operating concentrations of oligomycin, FCCP and rotenone/antimycin A. The measurement was performed: 4 × 3 cycle (24 min: 3 min mix; 2 min wait).

### Lactate and glucose measurement

To measure the glucose consumption and lactate production within the cell culture medium IPEC-J2 were grown on ThinCerts™ of 15 mm diameter, but medium was unaltered within the final 72 h of cultivation and treatment. After termination of cell culture duration cell culture medium was fully withdrawn from the cells and transferred into tubes separately accordingly to compartment distinguishing upper (apical) and lower (basolateral) compartment. All samples were stored on ice until measurement. Cell-free cell culture medium was used as blank. Glucose and lactate concentrations were determined immediately using Cobas C 501 (Roche, Germany) as well as reagents of test systems GLUC2 and LACT2 (Roche, Germany) respectively. The differences in glucose and lactate concentration between blank and samples were considered as glucose consumption and lactate production.

### ATP measurement

Cells were seeded on 12-well-ThinCerts for 10 days. On day 7, cells were treated apical or basolateral with different DON concentrations 50 ng/mL, 200 ng/mL, and 2000 ng/mL. On day 9, control cells were treated with or without carbonylcyanid-4-trifluormethoxyphenylhydrazon (FCCP, 5 or 10 µm, Sigma-Aldrich, Hamburg, Germany) for 24 h. On day 10, media was removed and a boiling hot puffer (300 µL/well; 100 mM TRIS; 4 mM EDTA; pH = 7.75, Roth, Karlsruhe, Germany) was added to the cells. In the next step, cells were scraped off the membrane with a cell scraper. The cell suspension was transferred into a tube, incubated for 2 min at 100 °C, centrifuged at 1000 × g for 60 s. Supernatants were pipetted into 96-well-microplate (50 µL/well; triplicates; Greiner bio-one; Frickenhausen, Germany). Samples were kept on ice until measurement. An ATP-standard curve was prepared according to manufacturer’s instructions (5 readings within 5 min; 25 °C; ATP Bioluminescence Assay Kit CLS II; Roche, Basel, Switzerland).

### Metabolic labeling and treatment of the cells

Labeling experiments were done according to Müller et al. (2015) in Met-free medium (c.c.pro, Germany) supplemented with either 4 mM Met (Sigma, Germany) or 4 mM AHA respectively. For that purpose, cells were treated as described above. On day 7 cells were incubated with different DON-concentrations for 48 h with DMEM/HAMF12 (see above). In the next step, medium was changed in all controls and treatment groups for medium without Met according to their treatment for 30 min. In the next step, all were changed to their according media (control or with DON) with AHA for 24 h. Furthermore, a second control was executed without treatment but with Met. At the end of the experiment, cells were removed with trypsin (10 min; 39 °C). Reaction was stopped with media + / − AHA or Met. Cells were centrifuged (10 min; 350 × g) and washed with PBS. Cell pellet was washed twice with PBS (pH 7.8) and frozen by − 80 °C.

#### Bioorthogonal non-canonical amino acid tagging (BONCAT)

Tagging of AHA labeled proteins was performed as essentially described in Dieterich et al. (2007) and Landgraf et al. (2015) (Dieterich et al. 2007; Landgraf et al. 2015). Briefly, cell pellets were lysed for 5 min at 95° in 300 µL 1 × PBS, pH 7.8, containing 0.2% Triton X100, 0.1% SDS, 1 × complete™ EDTA-free protease inhibitor cocktail (Roche) and 250 U/mL Benzonase^®^ nuclease. The resulting protein extracts were centrifuged for 5 min at 14.000 × g at 4 °C and the supernatant transferred into fresh 1.5 ml Eppendorf tubes. For click-chemistry samples were supplemented sequentially with 0.2 mM Triazole ligand (Tris[(1-benzyl-1*H*-1,2,3-triazol-4-yl)methyl]amine (TBTA), 25 µM biotin-PEO_3_-alkyne-tag, and 0.2 mg/mL copper(I)bromide-suspension (Acros), whereby the samples were thoroughly vortexed for 15 s between each supplementation-step. Subsequently samples were incubated under continuous agitation at RT. After 2 h reaction time precipitates were removed by centrifugation for 5 min at 3000 × g, 4 °C, and the remaining protein extracts further processed for quantification, SDS-PAGE and Western-blot.

#### Western-Blot experiments and quantitative analysis

For SDS-PAGE and Western Blot analysis protein fractions were solubilized with 4 × SDS sample buffer (250 mM Tris-HCl, pH 6.8, 1% SDS, 40% glycerol, 20% ß-mercaptoethanol, 0.004% bromophenol blue), boiled for 5 min (95 °C) and separated on 5–20% SDS–Polyacrylamide gradient gels, subsequently followed by transfer onto nitro cellulose membranes. After blotting, membranes were blocked with blocking solution (5% dry milk, 0.1% Tween 20 in 1 × TBS) for 1 h. Incubation with primary antibodies (anti-biotin, 1:10.000) was done at 4 °C over night in blocking solution. After intensive washing, blots were incubated for 90 min at room temperature with HRP-conjugated secondary antibodies (1:10.000) in blocking solution as well and finally developed with ECL reagent (Thermo Fisher Scientific) using the Odyssey^®^ Fc luminescence detector (LI-COR).

For normalization identical samples were separated by SDS-PAGE and stained for 1 h with 0.05% Coomassie brilliant blue dissolved in 50% methanol and 10% acetic acid, followed by distaining using a solution consisting of 5% methanol and 7% acetic acid. Determination and quantification of the Western-blot signals and Coomassie-stained gels was done using the Image Studio Lite version 5.0 software from LI-COR.

### Statistical analysis

Data are expressed as means with standard error. Data were tested on normal distribution with Shapiro–Wilk test and variance homogeneity (Levene-test). In the case of normal distribution and variance homogeneity, an ANOVA was performed with a Dunnett-Posthoc test (two-tailed; CON as control; (*p* ≤ 0.05*; *p* ≤ 0.01**; *p* ≤ 0.001***).

## Results

### Impact of DON on transepithelial resistance (TEER) of IPEC-1 cells

**Fig. 1:**
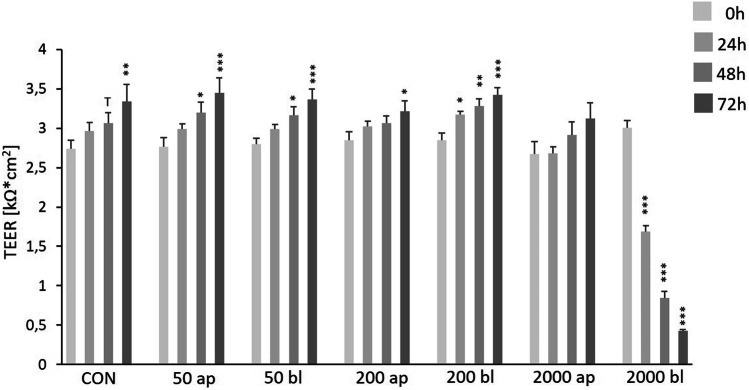
Impact of DON on transepithelial electrical resistance (TEER) in IPEC-1. Cells were grown on inserts and TEER was measured on day 10 (= 0 h/start of DON-application), 24 h, 48 h, and 72 h after DON-application (in ng/mL). TEER-values are expressed in k*cm^2^. Data are given as means (± SEM) of at least 8 experiments. We observed significantly increased TEER values in the CON-group (0 ng/mL DON) but also in the DON-treatment groups 50 ng/mL ap/bl and 200 ng/mL ap/bl after 48 h and 72 h of DON-application compared to the initial values of the corresponding insert (0 h). No differences were found in the group of 2000 ap compared to 0 h. In contrast, incubation of DON 2000 ng/mL bl resulted in significantly decreased TEER after 24 h and a value of 389 Ω*cm^2^ was observed after 72 h. (*p ≤ 0.05; **p ≤ 0.01; ***p ≤ 0.001)

Significantly increased TEER values were observed in the CON-group (0 ng/mL DON; Fig. 1) after 72 h but also in the DON-treatment groups 50 ng/mL ap/bl and 200 ng/mL ap/bl after 48 h and 72 h of DON-application compared to the initial values of the corresponding insert (0 h). No differences were found in the group of 2000 ap compared to 0 h. In contrast, incubation of DON 2000 ng/mL bl resulted in a significantly decreased TEER after 24 h and a value of 389 Ω*cm^2^ was observed after 72 h (***p ≤ 0.001).

### Effect of DON-concentration on oxygen consumption

**Fig. 2:**
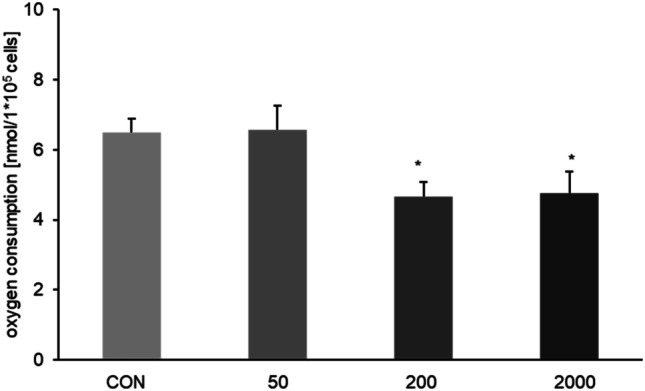
Oxygen consumption depends on DON concentration. Oxygen consumption was measured via fiber glass electrode in our Transwell^®^ system. A significantly decreased oxygen consumption was detected when DON 200 ng/mL [4.66 nmol/1*10^5^ cells; *p* ≤ 0.05] and DON 2000 ng/mL [4.77 nmol/1*10^5^ cells; *p* ≤ 0.05] was added independent of application site compared to CON [0 ng/mL; 5.66 nmol/1*10^5^ cells]. (*p ≤ 0.05; **p ≤ 0.01; ***p ≤ 0.001)

Our data on membrane-cultured cells were divided in 4 groups of one control and 3 DON treatment groups (50 ng/mL, 200 ng/mL, and 2000 ng/mL; Fig. 2) independent of apical or basolateral application. We observed a significant lower oxygen consumption in the treated groups of DON 200 ng/mL (4.66 nmol/1*10^5^ cells) and 2000 ng/mL (4.77 nmol/1*10^5^ cells) compared to control group (CON 0 ng/mL = 5.66 nmol/1*10^5^ cells).

### Effect of application on oxygen consumption

**Fig. 3:**
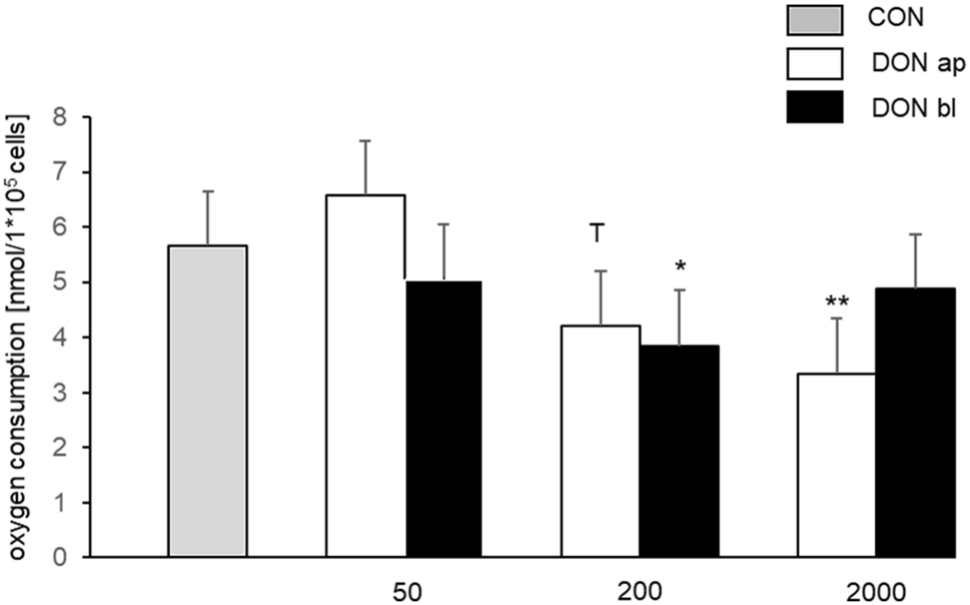
Oxygen consumption dependent on application site. A marked decrease was observed with 200 ng/mL [4.21 nmol/1*10^5^ cells; *p* = 0.086^ T^] but a significantly lower consumption was detected with 2000 ng/mL [3.34 nmol/1*10^5^ cells; *p* = 0.005] compared to CON (0 ng/mL), when DON was apically added. This effect was not confirmed for basolateral application for 2000 ng/mL treatment group but for 200 ng/mL [3.85 nmol/1*10^5^ cells; *p* ≤ 0.05]. (T = trend) (*p ≤ 0.05; **p ≤ 0.01; ***p ≤ 0.001)

All obtained data were separated in apical and basolateral application of DON (Fig. 3). For apically added DON a marked decrease was observed at 200 ng/mL (4.21 nmol/1*10^5^ cells; ANOVA; Dunnett, *p* = 0.086) but a significantly lower consumption was detected at 2000 ng/mL (3.34 nmol/1*10^5^ cells; ANOVA; Dunnett, *p* = 0.005) compared to control (CON 0 ng/mL; Fig. 3A). This effect could not be confirmed for basolateral application for 2000 ng/mL treatment group but for 200 ng/mL (3.85 nmol/1*10^5^ cells; ANOVA; Dunnett).

### Metabolic analysis via seahorse analyzer

**Fig. 4:**
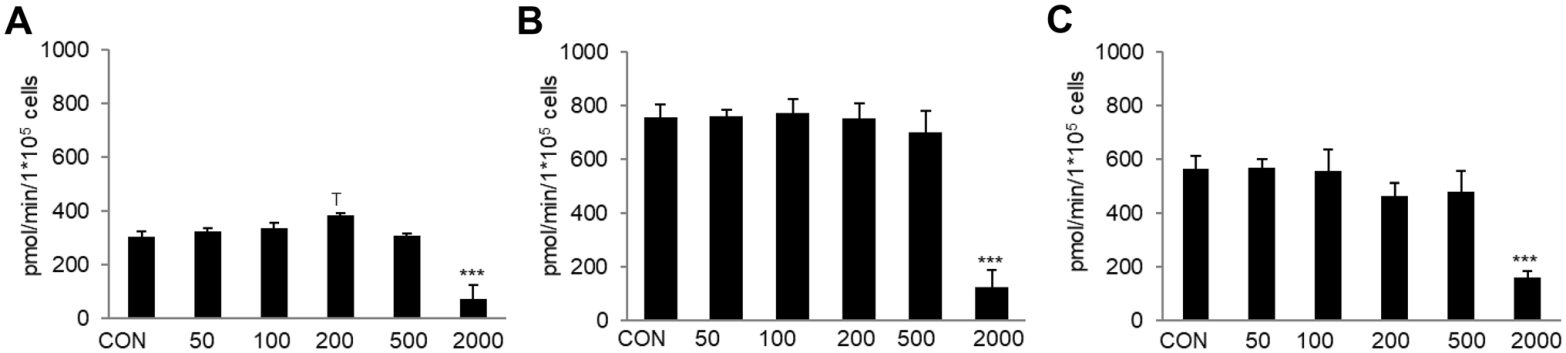
Analysis of metabolic function: basal respiration, maximal respiration and spare capacity. Metabolic function of IPEC-1 cells was analyzed via mitostress kit on a seahorse analyzer. **A** *Basal respiration:* at a concentration of 200 ng/mL DON, we observed an increase in the rate of basal oxygen consumption but a significant decrease at 2000 ng/mL DON (*p ≤ 0.05) compared to control. **B** *Maximal respiration*: Significantly lower values (***p ≤ 0.001) were found in the 2000 ng/mL treatment group in comparison to control. **C** *Spare capacity:* A treatment of 2000 ng/mL DON resulted in a decreased spare capacity (158.60 pmol/min/1*10^5^ cells; CON: 565.93 pmol/min/1*10^5^ cells). (*p ≤ 0.05; **p ≤ 0.01; ***p ≤ 0.001)

Cells were seeded on a plate to analyze metabolic function with a seahorse analyzer. *Basal respiration:* We observed a marked increase of the basal respiration rate in 200 ng/mL DON (384.17 pmol/min/1*10^5^ cells) and a significant decrease at 2000 ng/mL (71.82 pmol/min/1*10^5^ cells; *p* < 0.05) compared to control (CON 0 ng/mL = 303.56 pmol/min/1*10^5^ cells; Fig. 4A). *Maximal respiration:* In comparison to control (CON 0 ng/mL = 756.32 pmol/min/1*10^5^ cells), a significantly decreased maximal respiration was found at 2000 ng/mL (124.93 pmol/min/1*10^5^ cells; *p* < 0.001; Fig. 4B). *Spare capacity*: A treatment of 2000 ng/mL DON resulted in a decreased spare capacity (158.60 pmol/min/1*10^5^ cells; CON: 565.93 pmol/min/1*10^5^ cells; Fig. 4C).

**Fig. 5:**
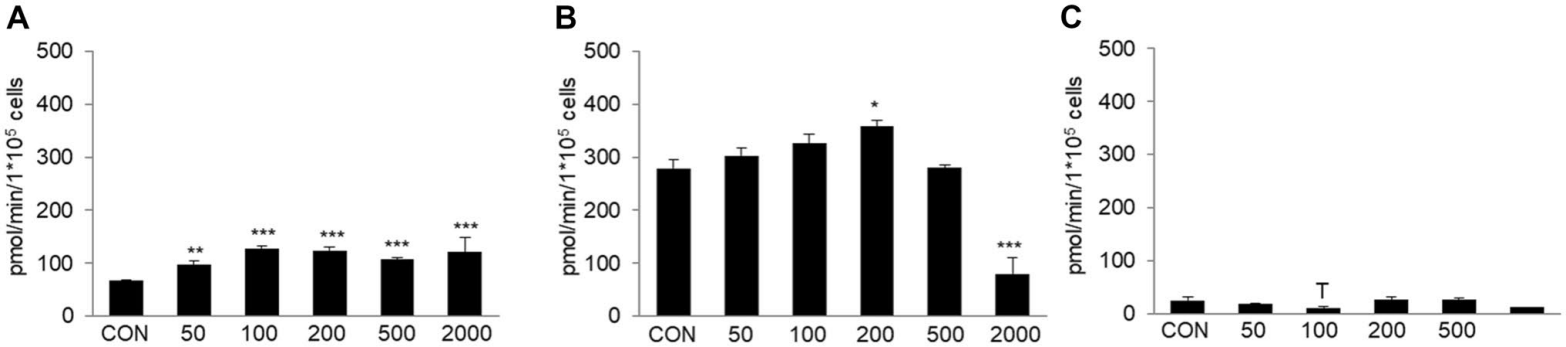
Analysis of metabolic function: non-mitochondrial oxygen consumption, ATP and proton leak. **A** *Non-mitochondrial oxygen consumption:* we detected a significantly increased non-mitochondrial oxygen consumption in all treatment groups compared to control (CON vs. DON 50 *p* ≤ 0.001; CON vs. all other groups: *p* ≤ 0.001). **B** *ATP:* a significantly increased ATP was detected in 200 ng/mL (*p* ≤ 0.05) and a significantly decreased was observed in 2000 ng/mL (*p* ≤ 0.001) compared to control **C** *Proton leak:* In the treatment group 100 ng/mL we observed a trend of a significantly lower proton leak in comparison to control. The treatment group 2000 ng/mL contains only one trail, as two trails could not be evaluated. (*p ≤ 0.05; **p ≤ 0.01; ***p ≤ 0.001)

*Non-mitochondrial oxygen consumption:* Furthermore, we detected a significantly increased non-mitochondrial oxygen consumption in all treatment groups compared to control (CON 0 ng/mL: 66.9 pmol/min/1*10^5^ cells, 50 ng/mL: 97.29 pmol/min/1*10^5^ cells (*p* = 0.01), 100: 128.45 pmol/min/1*10^5^ cells ng/mL (*p* < 0.001), 200 ng/mL: 124.14 pmol/min/1*10^5^ cells (*p* < 0.001); 500 ng/mL: 106.68 pmol/min/1*10^5^ cells (*p* = 0.001), and 2000 ng/mL:0.121 pmol/min/1*10^5^ cells (*p* < 0.001; Fig. 5A).

*ATP*: A significantly increased ATP was detected in 200 ng/mL (357.76 pmol/min/1*10^5^ cells) and a significantly decreased was observed in 2000 ng/mL (80.16 pmol/min/1*10^5^ cells) compared to control (CON 0 ng/mL: 278.1 pmol/min/1*10^5^ cells; Fig. 5B). *Proton leak*: In the treatment group 100 ng/mL (10.66 pmol/min/1*10^5^ cells) we observed a trend of a significantly lower proton leak in comparison to control (CON 0 ng/mL: 25.46 pmol/min/1*10^5^ cells; Fig. 5C).

### RNA expression in vivo and in vitro

In enterocytes of the DON-fed group we detected a significantly increased MHCII-RNA-expression (232.39%; *p* < 0.001) compared to CON-fed (100%; Table 2). Furthermore, an increased COX5B-RNA-expression (209.28%; *p* < 0.05) and increased Tricellulin-RNA-expression (201.68%; *p* < 0.05) were observed in the DON-fed group in comparison to CON-fed (100%). In IPEC-1, no changes could be detected with regard to COX5B, Tricellulin, GAPDH and TXNIP. A significant increase of PHB-RNA-expression was observed in DON 2000 ng/mL bl (317.57%; *p* < 0.01). In the same treatment group, we found a significantly lower SLC7A11-RNA-expression (31.79%; *p* < 0.05). A trend of an up-regulation of SLC7A11-RNA-expression was noticed in the treatment group DON-fed (215.74%) in vivo.

**Table 2:** Analysis of RNA expression in vivo and in vitro The in vivo RNA levels of the DON-fed group (concentration in ng/mL) were compared with the CON-fed group (= 100%). In the comparison of in vitro RNA levels, IPEC-1 CON group was used as reference and was set to 100%. (T = trend; nd = not detectable)

***Gene***	***Jejunal enterocyte*** ***(DON-fed vs. CON-fed)***	***IPEC-1*** ***50 ap***	***IPEC-1*** ***50 bl***	***IPEC-1*** ***200 ap***	***IPEC-1*** ***200 bl***	***IPEC-1*** ***2000 ap***	***IPEC-1*** ***2000 bl***
**GAPDH**	151.62	130.63	124.84	137.82	125.62	85.01	102.03
**COX5B**	209.28*	121.01	102.12	109.58	102.18	106.44	148.09
**MHCII**	232.39***	nd	nd	nd	nd	nd	nd
**PHB**	92.09	119.34	121.22	140.96	132.36	200.92	317.57**
**SLC7A11**	215.74^ T^	144.23	115.21	134.29	107.50	109.17	31.79
**TRIC**	201.68*	96.40	86.11	91.3	91.3	87.05	90.29
**TXNIP**	99.62	116.62	121.22	121.87	119.97	134.41	78.63

### Glucose consumption and lactate production

**Fig. 6:**
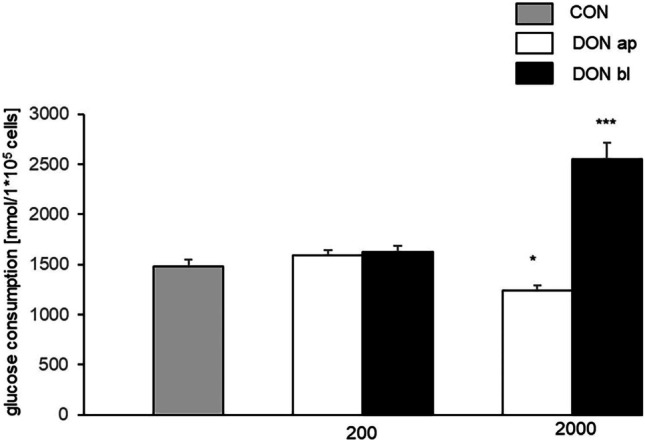
Glucose consumption. Total glucose consumption was determined over a period of 72 h (endpoint measurement). This resulted in a significantly lower glucose consumption when DON (2000 ng/mL) was added apically (1241.89 nmol/1*10^5^ cells; *p* = 0.05) and in a significantly higher consumption if DON (2000 ng/mL) attacked the cell from basolateral (2554.43 nmol/1*10^5^ cells; *p* ≤ 0.001***; CON: 1476.38 nmol/1*10^5^ cells). (*p ≤ 0.05; **p ≤ 0.01; ***p ≤ 0.001)

The total glucose consumption was analyzed in our IPEC1 cells over a period of 72 h (corresponds to DON treatment). We detected a significantly reduced glucose consumption (1241.89 nmol/1*10^5^ cells; *p* = 0.05*; Fig. 6A) when IPEC1 cells were treated apically with 2000 ng/mL DON compared to control (1476.38 nmol/1*10^5^ cells).

In contrast, a significantly higher glucose consumption was observed in the 2000 ng/mL bl treatment group (2554.43 nmol/1*10^5^ cells; *p* < 0.001***; Fig. 6B) compared to control.

**Fig. 7:**
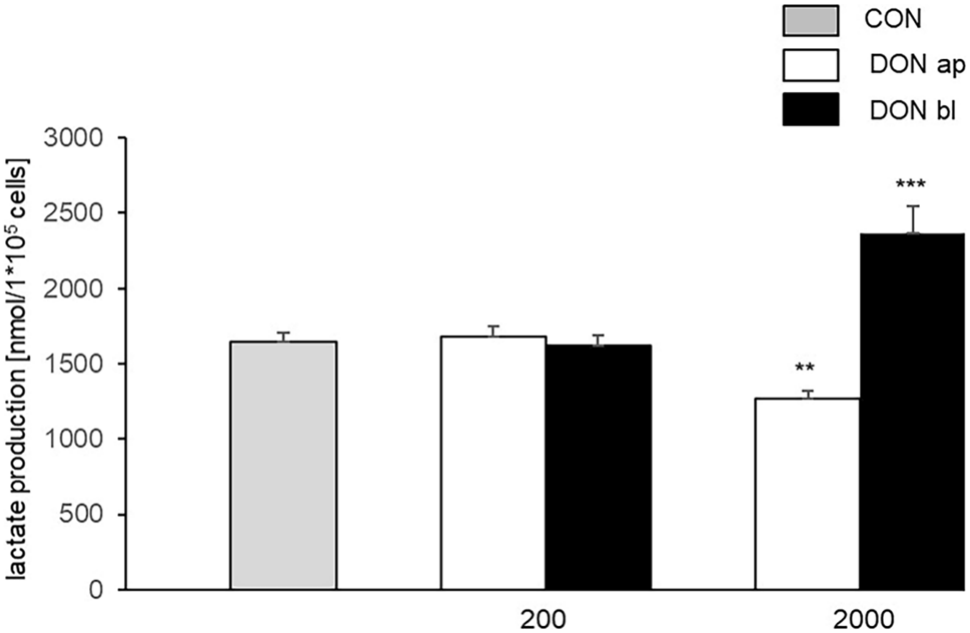
Lactate production. Like total glucose consumption, lactate production was examined over a period of 72 h using endpoint measurement. Similar to glucose consumption, a reduced lactate production was also observed when DON was added apically (2000 ng/mL ap DON: 1270.70 nmol/1*10^5^ cells; CON: 1643.06 nmol/1*105 cells; *p* = 0.004). Furthermore, significantly higher values were detected with DON 2000 ng/mL bl (2367.62 nmol/1*10^5^ cells) compared to control (CON). (*p ≤ 0.05; **p ≤ 0.01; ***p ≤ 0.001)

This is consistent with the lactate analysis. Here, we found a significantly lower lactate production of cells which were treated with 2000 ng/mL ap (1270.70 nmol/1*10^5^ cells; *p* = 0.004**; Fig. 7A) compared to control (1643.06 nmol/1*10^5^ cells). In contrast, a significant higher production was observed with DON 2000 ng/mL bl (2367.99 ng/mL; *p* < 0.001***; Fig. 7B).

### ATP content

**Fig. 8:**
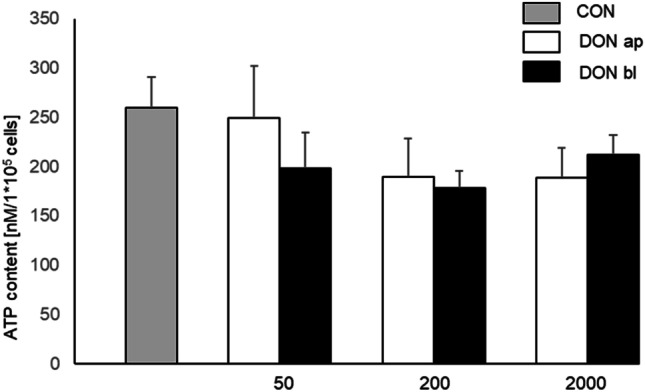
ATP content. ATP content was determined using a kit but no significant differences were found. However, lower values were found in cells, which were treated with DON from basolateral: CON 259.61 nM/1*10^5^ cells; 50 ap 249.23 nM/1*10^5^ cells; 50 bl 198.31 nM/1*10^5^ cells, 200 ap 189.59 nM/10^5^, 200 bl 178.19 nM/1*10^5^, 2000 ap 188.79 nM/1*10^5^ cells, and 2000 bl 212.33 nM/1*10^5^ cells

An ATP Bioluminescence Assay Kit was used for analysis of ATP content of DON-treated cells. We observed a slight but not significant decrease of ATP in our cells when they were treated with DON 200 ng/mL and 2000 ng/mL from apical or basolateral compared to control: *CON* 259.61 nM/1*10^5^ cells; *50 ap* 249.23 nM/1*10^5^ cells; *50 bl* 198.31 nM/1*10^5^ cells, *200 ap* 189.59 nM/10^5^, *200 bl* 178.19 nM/1*10^5^, *2000 ap* 188.79 nM/1*10^5^ cells, and *2000 bl* 212.33 nM/1*10^5^ cells (Fig. 8).

### Protein biosynthesis depends on DON-concentration and -application

**Fig. 9:**
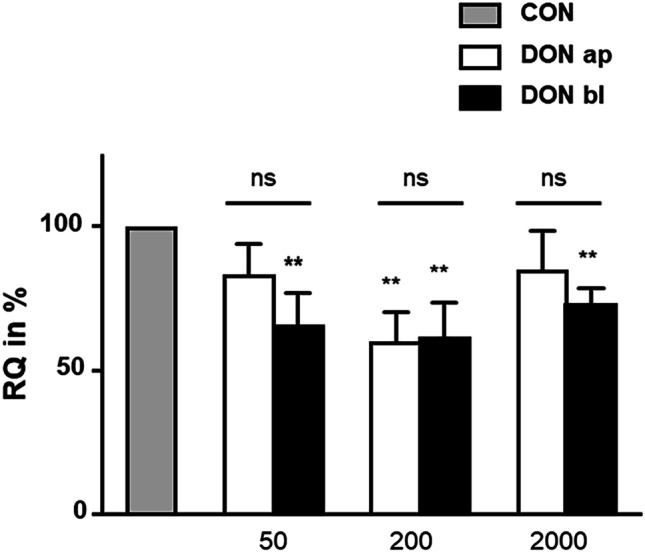
Analyses of protein biosynthesis (*N* = 3). After 72 h of DON treatment a significantly lower protein synthesis rate (RQ: relative quantification in %) was observed in 50 ng/mL bl, 200 ng/mL ap, 200 ng/mL bl, and 2000 ng/mL bl (*p* ≤ 0.01). No differences were found between the apical and basolateral application of DON. (*p ≤ 0.05; **p ≤ 0.01; ***p ≤ 0.001)

The control group was also treated with azide-bearing methionine surrogate azidohomoalanine (AHA) and used as reference value for the integration of AHA in comparison to all treatment groups (Fig. 9). After 72 h of DON-incubation, we detected a significantly lower protein biosynthesis in the treatment groups: 50 ng/mL bl, 200 ng/mL ap, 200 ng/mL bl, and 2000 ng/mL bl in comparison to control.

## Discussion

Deoxynivalenol is less toxic than T-2 toxin and many others like aflatoxins and patulin, but it is the most widely spread trichothecene and it is commonly found in barley, corn, rye, safflower seed, and wheat (Bennett and Klich 2003). Toxic effects of DON are well described with focus on immune system and gastrointestinal tract. The enterocytes of the intestinal barrier form a polarized monolayer, which separates the apical from the basolateral compartment. Tight junctions between adjacent cells represent an integral part of this compartmentalisation and any damage to them leads to an enhanced permeability of the cell layer and a decreased TEER which can lead to intestinal disorders. Pinton and colleagues detected significantly decreased TEER-values after 48 h of DON (5 µM = 1482 ng/mL) (Pinton et al. 2009), whereby it is not clear from which side (ap or bl) DON was added to the culture medium. This was confirmed by Diesing and co-workers. They found also decreased TEER-values combined with a lower ZO-1 and claudin-3 expression in IPEC-J2 cells, when cells were treated with DON from basolateral (Diesing et al. 2011).

Besides the loss of function of the intestinal barrier, important observations in the gastrointestinal tract are also thickening of the gut mucosa and changes in the villi and crypt morphology (Rotter et al. 1994; Awad et al. 2006; Dänicke et al. 2012). Based on this, it is not unreasonable to assume that DON influences the absorption of nutritional components and thus can also influence the metabolism of the enterocytes. In our study with IPEC-1 cells, we detected no effect on total glucose consumption under 50 and 200 ng/ml DON independent on application site, but a significantly lower glucose consumption in 2000 ng/mL DON-treatment group when DON attacks enterocytes from apical site. In contrast, we observed a significantly higher total consumption in 2000 ng/mL DON-treatment group when DON affects the cells from basolateral. This goes along with the data of total lactate production, where we found a significantly lower production, when DON was added to the apical compartment and a significantly increased production when DON was added to the basal compartment in the 2000 ng/mL treatment group. In a recent study of IPEC-J2, we observed the same effects of DON on glucose consumption and lactate production (Nossol et al. 2018). Glucose is taken up into epithelial cells predominantly by sodium-dependent glucose co-transporter SGLT1 (Wolffram et al. 2002; Wright et al. 2011). The indispensable role of SGLT-1 is confirmed by demonstrating the inability of animals lacking SGLT-1 to survive on a glucose-containing diet (Gorboulev et al. 2012). The efflux of glucose from enterocytes into the blood is thought to be mediated by GLUT2 as a facultative uniporter with a low affinity but high transport capacity (Mueckler et al. 1994). Bannert and co-workers have shown in DON-fed pigs (4.59 mg DON/kg feed) a lower glucose level at the portal sampling site at 30–45 min compared to control group, but they did not detect any increased or decreased lactate levels in pigs in the comparison of CON-fed and DON-fed groups in their study (Bannert et al. 2015).

In our study, we analyzed oxygen consumption with the focus on different DON-concentration. We detected in our study significantly lower oxygen consumption at 200 ng/ml and 2000 ng/ml when IPEC-1 cells were cultured on membranes (Fig. 2). In our seahorse analyzer, a DON-treatment of 200 ng/mL resulted in a trend of an increased oxygen consumption rate but at 2000 ng/mL in a significantly lower oxygen consumption rate compared to control. The difference in oxygen consumption rate may be due to the different cultivation methods. In the case of the seahorse experiment, cells were cultivated directly on plates and measured in a closed system. The measurements with fiber electrode were performed with cells cultured on membranes (Figs. 2 and 3). The disadvantage is that it is an open system, in which oxygen input can always take place through diffusion. Ngampongsa et al. found in their study the lowest oxygen consumption rate (OCR) at 0.78 µM (= 231 ng/ml) or higher concentrations of DON in cultured primary cardiomyocytes (Ngampongsa et al. 2013). They detected also a dose-dependent reduction of the mitochondrial reserve capacity (RC; 3.13 µM [= 918.08 ng/mL]) and hypothesized the property of cellular toxicity due to mitochondrial dysfunctions (Ngampongsa et al. 2013). Another important gene of mitochondria is PHB, also known as B-cell-receptor-associated protein 32 (BAP 32). PHB is an evolutionary conserved protein found in eukaryotic organisms (Lv et al. 2012) and occurs in normal cell membrane but also as an important protein of the mitochondria (Liemburg-Apers et al. 2011). In IPEC-1 cells a significantly increased PHB-mRNA-level was observed at DON 2000 ng/mL bl. In a previous study on IPEC-J2, we also found an up-regulation of PHB protein expression, when IPEC-J2 cells were incubated with 2000 ng/mL DON from apical or basolateral (Nossol et al. 2018). Latest studies describe PHB as “inner membrane mitophagy receptor” (Lahiri and Klionsky 2017) and contributes to mitochondrial quality control by removal of faulty mitochondria. PHB itself has a binding motif for LC3. LC3 is a key component of autophagy, the recycling system of the eukaryotic cell.

Furthermore, the expression of cytochrome C oxidase 5 (subunit A and B) is tightly regulated by oxygen (Burke et al. 1997). COX5A is switched off and COX5B is switched on when O_2_ concentration drops below a threshold of 0.5 µM O_2_. In vitro, we observed a marked increase at 2000 ng/mL bl DON and we also found a significant higher COX5B-RNA expression in DON-fed pigs. This could indicate a lower oxygen supply to the enterocytes in vivo rather than a poor supply of the whole tissue. Inflammation and oxygen levels are linked. Inflammation is often accompanied with hypoxia and hypoxia itself can cause inflammation. Oxygen levels vary between 0 and 19% in healthy mammalian tissue. The epithelium of the upper airways is close to the atmospheric oxygen level (approximately 19%) (Carreau et al. 2011). The oxygen levels of the gastrointestinal tract (GI) differ from the lumen to the lamina propria mucosae. The lumen is close to 0% oxygen and contains obligate anaerobic commensal bacteria (Albenberg et al. 2014). In the lamina propria, levels of 7% were found (Carreau et al. 2011). Immune cells like T cells and APC were observed in the lamina propria mucosae but also in the lamina epithelialis mucosae. In the present study, we observed significantly higher MHCII-RNA levels in DON-fed pig compared to CON-fed pigs. This could also indicate an inflammation in the tissue with migrated immune cells into the epithelia as shown by Nossol and co-workers (Nossol et al. 2013). On the other hand, it was shown that IECs were also able to express MHC class II molecules and can function as non-conventional APCs (Scott et al. 1980). IECs express MHCII under inflammatory conditions like in Inflammatory Bowel Disease IBD (Hirata et al. 1986), coeliac disease (Kelly et al. 1988) and graft-vs-host disease (GvHD) (Koyama et al. 2019), as well as during infections with Salmonella enterica or Heligomosomoides polygyrus. Moreover, a trend of an up-regulated SLC7A11 (cystine/glutamate antiporter) transporter was detected in DON-Fed pig. Cystine is reduced to cysteine, which is a rate-limiting precursor in glutathione synthesis. This is a process which protects cells from oxidative stress and is essential for proliferation, cell growth and metabolism. But also plays an important role in cancer establishment (Shi et al. 2021).

Furthermore, the application of DON independent of concentration resulted in an increase of non-mitochondrial oxygen consumption rate in vitro. One possible reason can be ROS production. This has already been shown for Deoxynivalenol. Kang and co-worker investigated the influence of DON on IPEC-J2 cells, and their analyses resulted in a dose-dependent increase of ROS at high concentration of DON (1000 ng/mL and 2000 ng/mL). In contrast, our study indicates that also low concentrations of DON, for example, 50 ng/mL can significantly increase ROS level in DON-treated cells. Furthermore, they increase lipid peroxidation, and this will lead to a single-strand break in DNA (Chaudhari et al. 2009). Oxidative stress is importantly involved in protein synthesis inhibition (Dänicke et al. 2006; Pestka and Amuzie 2008). In vivo only a few studies exist that analyzed the effect of DON on protein synthesis. Robbana-Barna et al. used pure DON at experimental single doses of 4, 10, 20 and 80 mg/kg (Robbana-Barna et al. 1987). In contrast, Azcona-Olivera took 5 and 25 mg/kg live weight which was given orally (Azcona-Olivera et al. 1995). Dänicke et al. investigated in their study the effect of DON on protein synthesis in pigs. They showed in their study that the fractional synthesis rate (FSR) was significantly reduced in ileum of DON-exposed pigs but not in jejunum or duodenum (Dänicke et al. 2006). In our study, we could document that DON inhibits the protein synthesis of newly synthesized proteins in vitro about 50% of control at a concentration of 50 ng/mL bl, 200 ng/mL ap and bl, and in the 200 ng/mL bl treatment group. In contrast, IPEC-J2 cells don not show an inhibition of protein synthesis but a significant increasing of protein biosynthesis (Nossol et al. 2018).

The in vivo situation can be well mapped with IPEC-1 regarding to the reduced protein biosynthesis with DON treatment. As well as IPEC-J2, these cells showed no reduction in protein biosynthesis (Nossol et al. 2018) as in vivo. Further studies on protein biosynthesis in these apparently different regulated cell lines under DON-treatment have to be performed and might allow conclusion on the in vivo situation in jejunum and ileum. In addition, Deoxynivalenol influences indirectly the metabolic state of the cells due to a decreased glucose uptake, decreased lactate production and decreased basal respiration rate when DON attacks the cells from apical at high DON concentration. In contrast, a basolateral addition of high DON concentration results in an increased glucose uptake and lactate production. These results need to be investigated by further experiments with the Seahorse analyzer and DON-treated cells cultured on membranes not on plate-seeded cells.

## Data Availability

The datasets generated during and/or analysed during the current study are available from the corresponding author on reasonable request.

## Abbreviations

AHA: Azidohomoalanine
BONCAT: Biorthogonal non-canonical amino acid tagging
cDNA: Complementary DNA
CD16: Cluster of differentiation
CON: Control
COX5B: Cytochrome C oxidase 5 subunit B
DMDC: Dimethyldicarbonate
DON: Deoxynivalenol
EDTA: Ethylenediaminetetraacetate
EGF: Epidermal growth factor
FBS: Fetal bovine serum
FCCP: Carbonylcyanid-4-trifluormethoxyphenylhydrazon
FSR: Fractional synthesis rate
GAPDH: Glyceraldehade-3-phosphate dehydrogenase
HEPES: 16 MM 4-(2-hydroxyethyl)-1-piperazineethansulfonic acid
IL-1β: Interleukin 1β
IL-6: Interleukin 6
IBD: Inflammatory bowel disease
IPEC-1: Intestinal porcine epithelial cells (ileal and jejunal)
IPEC-J2: Intestinal porcine epithelial cells (jejunal)
ITS: Insulin-transferrin-selenium
KEGG: Kyoto Encyclopedia of Genes and Genomes
LPS: Lipopolysaccharides
Met: Methionine
MHCII: Major human histocompatibility complex class II
NIV: Nivalenol
PBS: Phosphate buffered saline
PHB: Prohibitin
RT: Room temperature
SDS: Sodium dodecal sulfate
SLC7A11: Solute carrier family 7 member 11
TBS: TRIS buffered saline
TBTA: Tris[(1-benzyl-1*H*-1,2,3-triazol-4-yl)methyl]amine
TEER: Transepithelial electrical resistance
TNFα: Tumor necrosis factor α
TXNIP: Thioredoxin interacting protein
